# Anesthesia-Induced Hypothermia Attenuates Early-Phase Blood-Brain Barrier Disruption but Not Infarct Volume following Cerebral Ischemia

**DOI:** 10.1371/journal.pone.0170682

**Published:** 2017-01-24

**Authors:** Yu-Cheng Liu, Yu-Da Lee, Hwai-Lee Wang, Kate Hsiurong Liao, Kuen-Bao Chen, Kin-Shing Poon, Yu-Ling Pan, Ted Weita Lai

**Affiliations:** 1 Graduate Institute of Clinical Medical Science, China Medical University, Taichung, Taiwan; 2 Department of Anesthesiology, China Medical University Hospital, Taichung, Taiwan; 3 Graduate Institute of Biomedical Sciences, China Medical University, Taichung, Taiwan; 4 Translational Medicine Research Center, China Medical University Hospital, Taichung, Taiwan; Hungarian Academy of Sciences, HUNGARY

## Abstract

Blood-brain barrier (BBB) disruption is thought to facilitate the development of cerebral infarction after a stroke. In a typical stroke model (such as the one used in this study), the early phase of BBB disruption reaches a peak 6 h post-ischemia and largely recovers after 8–24 h, whereas the late phase of BBB disruption begins 48–58 h post-ischemia. Because cerebral infarct develops within 24 h after the onset of ischemia, and several therapeutic agents have been shown to reduce the infarct volume when administered at 6 h post-ischemia, we hypothesized that attenuating BBB disruption at its peak (6 h post-ischemia) can also decrease the infarct volume measured at 24 h. We used a mouse stroke model obtained by combining 120 min of distal middle cerebral arterial occlusion (dMCAo) with ipsilateral common carotid arterial occlusion (CCAo). This model produced the most reliable BBB disruption and cerebral infarction compared to other models characterized by a shorter duration of ischemia or obtained with dMCAO or CCAo alone. The BBB permeability was measured by quantifying Evans blue dye (EBD) extravasation, as this tracer has been shown to be more sensitive for the detection of early-phase BBB disruption compared to other intravascular tracers that are more appropriate for detecting late-phase BBB disruption. We showed that a 1 h-long treatment with isoflurane-anesthesia induced marked hypothermia and attenuated the peak of BBB disruption when administered 6 h after the onset of dMCAo/CCAo-induced ischemia. We also demonstrated that the inhibitory effect of isoflurane was hypothermia-dependent because the same treatment had no effect on ischemic BBB disruption when the mouse body temperature was maintained at 37°C. Importantly, inhibiting the peak of BBB disruption by hypothermia had no effect on the volume of brain infarct 24 h post-ischemia. In conclusion, inhibiting the peak of BBB disruption is not an effective neuroprotective strategy, especially in comparison to the inhibitors of the neuronal death signaling cascade; these, in fact, can attenuate the infarct volume measured at 24 h post-ischemia when administered at 6 h in our same stroke model.

## Introduction

The blood-brain barrier (BBB) protects the brain from circulating plasma proteins and inflammatory cells. Therefore, disturbances of the BBB are thought to contribute to the pathogenesis of many neurodegenerative diseases, and interventions that inhibit BBB disruption are being developed to hinder disease progression. In particular, BBB disruption is a hallmark of ischemic stroke [1–3]. Ischemic insult to either the endothelial cells forming the BBB or the supportive cells near the capillary beds, which include the glia and pericytes, can contribute to barrier disruption. Consistent with the notion that BBB disruption contributes to neurodegeneration, the degree of BBB disruption strongly predicts the severity of neuronal injury within the first 3 weeks after an ischemic stroke [1, 3–5]. Moreover, some drugs that can mitigate ischemic BBB disruption have been shown to also reduce the volume of cerebral infarction after a stroke [6, 7].

BBB disruption following transient focal cerebral ischemia is a biphasic process. The early phase begins after reperfusion, reaches its peak approximately 6 h after the onset of cerebral ischemia, and recovers, mostly or completely, in the successive 8–24 h [1, 8–11]. This early phase is caused primarily by an increase in endothelial transcytosis, characterized by little or no degradation of tight junction [10, 12], and resulting in a marked cerebral extravasation of plasma albumin and related intravascular tracers [1, 8–14] such as the Evans blue dye (EBD) [1, 8, 9, 11, 13, 14]. Interestingly, the augmented transcytosis that occurs in the early phase of BBB disruption does not result in a noticeable increase in extravasation of small molecules or immunoglobulins [10]. In marked contrast, the late phase of BBB disruption begins 48–58 h following cerebral ischemia [8, 10] and is associated with the disruption of tight junction integrity and increased transcellular and paracellular transport of many intravascular tracers [10]. It should be noted that the nature of BBB disruption depends on the chosen stroke model and, for this reason, different time courses and cellular mechanisms of BBB disruption have also been reported [2, 5, 15].

Brain infarction following focal cerebral ischemia develops in the first 24 h post-ischemia, when the first phase of BBB disruption has recovered but the second phase has not yet begun. Given that therapeutic interventions administered as late as 6 h post-ischemia has been shown to reduce infarct volume in different animal models [16, 17], we asked whether brief anesthesia-induced hypothermia at the same time point, corresponding to the peak of early-phase BBB disruption, hinders ischemic BBB disruption and/or reduces infarct volume. To answer this question, we utilized a model of cerebral ischemia that has previously shown infarct volume reduction following the administration of an inhibitor of the neuronal death signaling cascade 6 h post-ischemia [17]. Because early-phase BBB disruption is characterized by the selective leakage of albumin but not of other intravascular tracers [10], BBB permeability was quantified by measuring the cerebral extravasation of the albumin-binding tracer EBD. In fact, with a cerebral extravasation property that is distinct from that of a non-albumin-binding tracer [18], EBD is able to extravasate in the early phase of BBB disruption [1, 8, 9, 11, 13].

The lack of clinical advancement despite much basic stroke research is a subject of active discussion and debate amongst stroke researchers, and many reasons have been proposed to explain the continued failure of experimental treatments or interventions in stroke trials despite their reported effectiveness in preclinical animal models [19–21]. The lack of adherence to animal research guidelines, poor reporting of preclinical experimental procedures, and under-reporting of negative findings are some of the primary obstacles that can prevent the translation of stroke research [21]. Such endeavors not only lead to unnecessary repetition of animal experiments and premature execution of clinical trials, but also dissuade pharmaceutical companies to further invest in translational stroke research and stroke clinical trials [21]. Here, we report the lack of neuroprotection when the peak of early-phase BBB disruption following stroke was inhibited by anesthesia-induced hypothermia. In accordance to ARRIVE recommendations, the treatment groups were randomized and blinded to the investigators carrying out the animal experiments.

## Materials and Methods

### Animals

Male C57BL/6 mice (21–27 g) purchased from the National Laboratory Animal Center (Taipei) were used in this study. They were housed in large-diameter cages in groups of 10 per cage and had free access to standard rodent chow and water. Room lighting was controlled under a 12:12 h light/dark cycle, and the experiments were always performed during the light cycle. The experimental protocols were carried out in accordance with the ARRIVE guidelines and the Institutional Guidelines of the China Medical University for the Care and Use of Experimental Animals (IGCMU-CUEA). All the procedures were approved by the Institutional Animal Care and Use Committee (IACUC) of the China Medical University (Taichung, Taiwan) (Protocol No. 103-224-NH).

### Cerebral ischemia

To induce focal ischemia, the mice were anesthetized with pentobarbital (65 mg/kg, i.p.) or tiletamine/zolazepam (50 mg/kg each, i.p.), and subjected to distal middle cerebral arterial occlusion (dMCAo) with common carotid arterial occlusion (CCAo) using the protocol described by Chen et al [22]. In brief, the right dMCA was ligated with a 10–0 nylon suture, and the right CCA was occluded using a non-traumatic arterial clip. After inducing ischemic periods of various lengths (30–120 min), the nylon suture and the arterial clip were removed to allow complete reperfusion. Body temperature was maintained at ~37°C throughout the procedure by means of a heating pad and 0.5% bupivacaine was preemptively administered first subcutaneously and then topically at the sites of surgery to achieve analgesia. The mice were allowed to recover in their home cages after reperfusion.

To determine BBB permeability, mice received EBD (4% solution in saline; 2 ml/kg) via tail vein injection under very brief isoflurane-anesthesia 6 h after the onset of ischemia. In our experience, this time point is associated with the greatest increase in EBD extravasation following dMCAo/CCAo. After allowing a 1 h-long period for EBD extravasation, the mice were euthanized under urethane-anesthesia and perfused with saline to clear the circulation of EBD. The concentration of EBD extravasated into each hemisphere was quantified as previously described [23].

To quantify infarct volume, mice were euthanized with an overdose of urethane (4 g/kg, i.p.) 24 h after the onset of ischemia, and coronal sections of their isolated brains were stained with 2,3,5-triphenyltetrazolium chloride (TTC) as previously described [24]. Infarct volume was then quantified with the image analysis software Image J.

**Fig 1:**
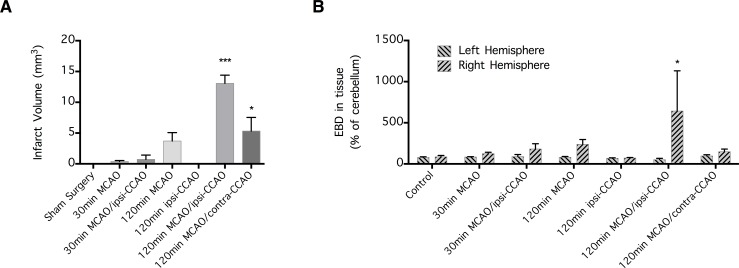
Early infarct volume and blood-brain barrier (BBB) disruption following distal middle cerebral arterial occlusion (dMCAo). Mice were subjected to 30 min or 120 min of cerebral ischemia induced by dMCAo with or without ipsilateral (ipsi-) or contralateral (contra-) common carotid arterial occlusion (CCAo). (**A**) Infarct volume was revealed by 2,3,5-triphenyltetrazolium chloride staining 24 h post-ischemia (n = 6 per group). Data are expressed as mean ± SEM, and *p<0.05 and ***p<0.001 indicate a significant infarct volume. (**B**) BBB permeability was determined by measuring Evans blue dye (EBD) extravasation 6 h post-ischemia (n = 3–5 per group). Data are expressed as mean ± SEM, and *p<0.05 indicates a significant difference compared to the contralateral hemisphere.

**Fig 2:**
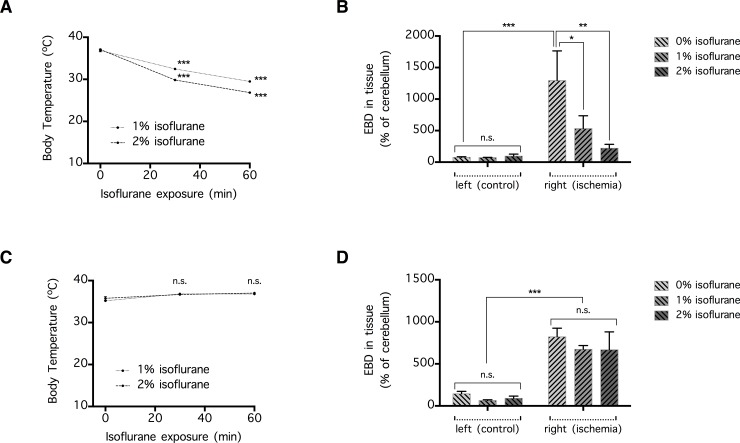
Hypothermic but not normothermic isoflurane-anesthesia attenuates early blood-brain barrier (BBB) disruption following distal middle cerebral arterial occlusion (dMCAo). (**A**) Body temperature was measured continuously in mice using a rectal probe, and recorded at 0, 30, and 60 min after exposure to 1% or 2% isoflurane (n = 5 per group). Data are expressed as mean ± SEM, and ***p<0.001 indicates a significant change in body temperature over time. (**B**) To induce ischemic BBB disruption, the mice were subjected to 120 min of dMCAo coupled with ipsilateral common carotid arterial occlusion. At 4 h post-reperfusion (6 h after the onset of ischemia), the mice were placed for 1 h inside a gas chamber filled with 0%, 1%, or 2% isoflurane to induce hypothermia. The severity of BBB disruption was determined by comparing the concentrations of intravascular tracer Evans blue dye (EBD), injected 4 h post-reperfusion and quantified 1 h thereafter, extravasated into the left (control, non-ischemic) and the right (ischemic) hemisphere (n = 13–14 per group). Data are expressed as mean ± SEM. *p<0.05 and ***p<0.001 indicate a significant difference, whereas n.s. indicates no significant difference. (**C**) Same as (A), except that body temperature was maintained with a heating pad (n = 4 per group). (**D**) Same as (B), except that body temperature was maintained with a heating pad (n = 4–5 per group).

**Fig 3:**
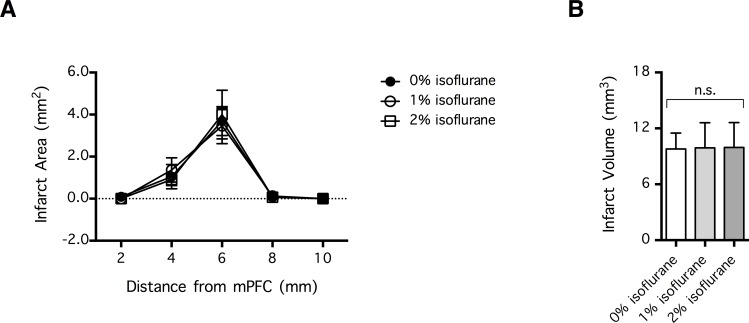
Hypothermia induced by isoflurane-anesthesia had no effect on early infarct volume following distal middle cerebral arterial occlusion (dMCAo). To induce brain infarction, mice were subjected to 120 min of dMCAo coupled with ipsilateral common carotid arterial occlusion. At 4 h post-reperfusion (6 h after the onset of ischemia), hypothermia was induced by exposure to 1% or 2% isoflurane for 1 h. Control animals received 0% isoflurane in the same gas chamber and were normothermic. (**A**) Infarct area per coronal section was revealed by 2,3,5-triphenyltetrazolium chloride staining 24 h post-ischemia (n = 10 per group). Data are expressed as mean ± SEM. The x-axis indicates the location of the coronal section relative to the medial prefrontal cortex (mPFC). No significant difference was detected. (**B**) Infarct volume derived from (A); n = 10 per group. Data are expressed as mean ± SEM. n.s. indicates no significant difference.

A non-blinded pilot study was initially performed to determine the optimal dMCAo/CCAo protocol for inducing brain infarction and BBB disruption (Fig 1A and 1B). In this pilot study, infarct volumes were measured 24 h after the onset of ischemia and the results obtained from different groups of mice were compared. These groups included mice subjected either to sham surgery or different lengths of ischemic periods induced as follows: 30 min dMCAo, 30 min dMCAo + ipsilateral-CCAo, 120 min dMCAo, 120 min right-CCAo, 120 min dMCAo + ipsilateral-CCAo, and 120 min dMCAo + contralateral-CCAo (n = 6 per group) (Fig 1A). Similarly, the BBB permeability to EBD 6 h post-ischemia was compared in control mice (n = 5) and mice subjected to 30 min dMCAo (n = 4), 30 min dMCAo + ipsilateral-CCA occlusion (CCAo) (n = 3), 120 min dMCAo (n = 4), 120 min right-CCAo (n = 4), 120 min dMCAo + ipsilateral-CCAo (n = 4), and 120 min dMCAo + contralateral-CCAo (n = 4) (Fig 1B). Based on the results of these experiments, an ischemic period of 120 min induced by dMCAo + ipsilateral-CCAo was selected as the optimal protocol for the subsequent experiments (Figs 2 and 3), which were performed in a systematic, randomized, and blinded manner.

### Hypothermia induced by isoflurane inhalation

To induce hypothermia, we used doses of isoflurane known to have little or no effect on blood gas and blood pressure [25, 26]. In addition, a similar degree of hypothermia has been shown to have no effect on intra- and post-ischemic cerebral blood flow [27]. To confirm that isoflurane-anesthesia produces hypothermia, a subset of mice was briefly anesthetized by means of a quick exposure to air carrying 4% isoflurane, to facilitate the placement of a standard mouse rectal probe. The probe was securely taped against the mouse’s tail so that body temperature could be monitored in a continuous manner while the mouse was recovering from the anesthesia. To examine the effect of isoflurane on body temperature, each mouse was allowed to recover for 1 h in a gas chamber (room temperature of ~23°C) containing air carrying either 1% isoflurane (n = 5) or 2% isoflurane (n = 5). During this time, the mouse’s body temperature was monitored and recorded (Fig 2A). In a control mouse that recovered in a gas chamber without isoflurane, this protocol did not produce hypothermia.

To examine the effects of hypothermia on ischemic BBB disruption, mice were subjected to a 2 h-long dMCAo/CCAo procedure, and 6 h after the onset of ischemia, they received EBD administration and were placed for 1 h inside a gas chamber containing one of the followings: (1) air (n = 14), (2) air carrying 1% isoflurane (n = 14), or (3) air carrying 2% isoflurane (n = 13). Thereafter, the amount of EBD that extravasated into the brain parenchyma was quantified (Fig 2B). In addition, to examine whether hypothermia had an effect on cerebral infarction, mice were subjected to a 2 h-long dMCAo/CCAo procedure, and 6 h after the onset of ischemia, they were placed for 1 h inside a gas chamber containing one of the followings: (1) air (n = 10), (2) air carrying 1% isoflurane (n = 10), or (3) air carrying 2% isoflurane (n = 10). Subsequently, they were returned to their home cage to recover from the anesthesia-induced hypothermia, and their brain infarct volumes were quantified 24 h after the onset of ischemia (Fig 3A and 3B). To determine whether isoflurane had an effect on blood electrolyte concentration, blood samples were collected from a control mouse and mice treated with 1% or 2% isoflurane (n = 1 each) and analyzed with a blood electrolyte analyzer (Stat Profile Critical Care Xpress, Nova Biomedical), as previously described [28].

To examine whether normothermia could be maintained during isoflurane-anesthesia by means of a heating pad, a subset of mice was subjected to rectal probe placement under brief isoflurane-anesthesia. Afterward, each mouse was allowed to recover for 1 h on a heating pad inside the gas chamber, which contained air carrying either 1% isoflurane (n = 4) or 2% isoflurane (n = 4) (Fig 2C). The heating pad received feedback from the rectal probe, and automatically adjusted heating temperature in accordance to measured body temperature. To examine the effect of normothermic isoflurane on ischemic BBB disruption, mice were subjected to 2 h of dMCAo/CCAo, and 6 h after the onset of ischemia, they received EBD administration and rectal probe-placement as described above, and were placed for 1 h inside a gas chamber containing the rectal probe-coupled heating pad and one of the followings: (1) air (n = 5), (2) air carrying 1% isoflurane (n = 4), or (3) air carrying 2% isoflurane (n = 4). Thereafter, the amount of EBD that extravasated into the brain parenchyma was quantified (Fig 2D).

### Randomization and exclusion

Unless otherwise indicated, the experiments involving cerebral ischemia were performed in a randomized and blinded manner. Hence, the investigators performing the dMCAo/CCAo surgery, injecting EBD, collecting brain tissue, quantifying BBB permeability, and measuring cerebral infarction area/volume were unaware of the treatment groups (control vs. 1% isoflurane vs. 2% isoflurane). To avoid randomization bias, treatment groups were randomized using the = RANDBETWEEN(1, 3) function in Microsoft Excel. A sample size of 4–5 was used in most experiments, as this sample size was sufficient for detecting significant BBB disruption in our pilot study. To further ensure reproducibility of the key findings, a larger sample size was used in the experiments relating to the effect of hypothermia on BBB permeability (n = 13–14) and infarction (n = 10). No animals were excluded from data analysis, and the n = 1 difference in sample size between some treatment groups was due to animal availability at the time of the experiment.

### Statistical analysis

Data were expressed as the mean ± SEM. EBD extravasation into each hemisphere or brain region was compared using 2-way repeated-measures ANOVA (i.e., different brain regions from the same animal were matched), whereas infarct volumes were compared using 1-way ANOVA. In both cases, significant differences were confirmed with the Sidak’s multiple comparisons test. Changes in body temperature over time were compared using 2-way repeated-measures ANOVA (i.e., different time points from the same animal were matched) followed by the Sidak’s multiple comparisons test.

## Results

### Cerebral ischemia

To establish a model of cerebral ischemia characterized by both a robust BBB disruption and a sizable infarct volume, mice were subjected to a number of dMCAo/CCAo protocols that produced different degrees of cerebral infarction (see Methods). Of all the protocols, 120 min of ischemic time induced by dMCAo + ipsilateral-CCAo produced the greatest infarct volume (n = 6 per group; p<0.001) (Fig 1A). Compared to other protocols, this method also produced the greatest unilateral increase in BBB permeability, as indicated by the cerebral extravasation of the intravascular tracer EBD (n = 3–5 per group; p<0.05) (Fig 1B). Conversely, 120 min of ischemic time induced by dMCAo + contralateral-CCAo produced a moderate infarct volume (p<0.05) with a disruption of the BBB that was only mildly noticeable (p>0.05) (Fig 1A and 1B). These results were consistent with previous reports showing that moderate cerebral ischemia causes neuronal injury but not BBB disruption [2]. In comparison, neither 30 min dMCAo nor 30 min dMCAo + ipsilateral-CCAo resulted in cerebral infarction or a significant BBB disruption (Fig 1A and 1B). This result confirms that our surgical procedure, even when associated with a mild ischemic insult, does not cause cerebral infarction or affect BBB permeability.

### Hypothermia induced by isoflurane inhalation

Next, we investigated whether hypothermia induced by isoflurane-anesthesia can reduce early-phase BBB disruption, which, in our experience and as reported by others [10], reaches a peak 6 h after the onset of cerebral ischemia and recovers in the successive 24 h. As expected, mice subjected to isoflurane-anesthesia experienced dose-dependent hypothermia, as indicated by a decrease in rectal temperature from 37.0 ± 0.2°C to 29.5 ± 0.2°C (with 1% isoflurane) and to 26.8 ± 0.2°C (with 2% isoflurane) over a period of 1 h (n = 5 per group; p<0.001 compared to other time points) (Fig 2A). When administered at 6 h post-ischemia, this hypothermic regimen attenuated ischemic BBB disruption in a dose-dependent manner, as indicated by the parenchymal concentration of the intravascular tracer EBD extravasated into the ipsilateral (ischemic) hemisphere (n = 13–14 per group) (p<0.05 for 1% isoflurane and p<0.01 for 2% isoflurane) (Fig 2B). It should also be noted that isoflurane-induced hypothermia had no effect on basal BBB permeability, as shown by the concentration of EBD extravasated into the contralateral (non-ischemic) hemisphere (p>0.05) (Fig 2B). In a subset of mice (n = 1 for control, 1% isoflurane, and 2% isoflurane), blood electrolyte analysis showed that isoflurane treatment had no effect on blood electrolyte concentration or osmolarity (data not shown). To determine whether the effect of isoflurane-induced hypothermia could be due to other effects of isoflurane, we exposed the mice to the same isoflurane-treatment in presence of a heating pad, which maintained the mouse rectal temperature at 36.8 ± 0.3°C (with 1% isoflurane) or 37.0 ± 0.3°C (with 2% isoflurane) throughout the 1 h-long treatment period (n = 4 per group; p>0.05 compared to other time points) (Fig 2C). Under this normothermic condition, isoflurane-anesthesia had no effect on ischemic or basal BBB permeability, as indicated by the concentrations of EBD extravasated into the ipsilateral and contralateral hemispheres, respectively (n = 4–5 per group) (p>0.05 for either 1% or 2% isoflurane) (Fig 2D).

To investigate whether inhibition of early-phase BBB disruption at its peak (6 h post-ischemia) can decrease acute infarct volume, mice were administered isoflurane-anesthesia at 6 h post-ischemia and then subjected to the associated hypothermia for 1 h. Despite the obvious inhibitory effect on the peak of early-phase BBB disruption (Fig 2B), isoflurane-induced hypothermia at 6 h after the onset of ischemia had no effect on the volume of cerebral infarction, indicated by the TTC staining of coronal brain sections performed at 24 h post-ischemia (n = 10 per group) (p>0.05) (Fig 3A and 3B).

## Discussion

Hypothermia is well-known to exert powerful neuroprotective effects [29–33], but its clinical use in the management of stroke remains mostly limited to the treatment of increased intracranial pressure and cerebral edema rather than being used for neuroprotection [34, 35]. Like most neuroprotective treatments, intra-ischemic hypothermia is more neuroprotective than post-ischemic hypothermia [30]. In fact, hypothermia has already shown neuroprotective efficacy in patients with global cerebral ischemia caused by cardiac arrest and neonatal asphyxia, for which intra-ischemic treatment is possible [32, 33, 36]. However, stroke patients are typically admitted to the hospital several hours following the onset of focal ischemia, making intra-ischemic hypothermia impractical. In line with the known therapeutic effect of hypothermia against cerebral edema following focal ischemia [35], hypothermia is also recognized to be effective at preventing ischemic BBB disruption [37]. Given the presumed role of the BBB in protecting the brain from circulating neurotoxins and inflammatory cells, we asked whether a delayed protection of the BBB by hypothermia at a critical period, when stroke-induced BBB disruption reaches its peak, may protect the brain against infarction within a clinically manageable time window.

Anesthesia is an effective method for inducing hypothermia, and hypothermia induced by isoflurane-anesthesia has been shown to alter hippocampal protein expression and brain function independently of other anesthesia-related effects [38]. Moreover, neurobiological changes induced by isoflurane-anesthesia can be achieved at doses that do not significantly alter blood gas and blood pressure [25, 26]. In this study, we further showed that isoflurane-anesthesia induced dose-dependent hypothermia without affecting blood electrolyte concentration and osmolarity. Interestingly, although hypothermia decreased post-ischemic cerebral vascular permeability in this study, decrease in brain temperature has been shown to have little effect on post-ischemic cerebral blood flow [27]. Given that the early phase of ischemic BBB disruption is characterized primarily by an increase in endothelial transcytosis rather than a deficit in tight junction integrity [10], hypothermia induced by isoflurane-anesthesia probably hindered plasma protein extravasation at this time point by impairing vesicular formation and trafficking. Notably, a recent study has shown that isoflurane is able to prevent BBB disruption induced by subarachnoid hemorrhage in mice [39]. Unfortunately, body temperature of the mice was not measured in that study. Therefore, it remains unclear whether the reported effect was due to isoflurane-induced hypothermia or rather non-hypothermic effects of isoflurane [39]. In the present study, we showed that isoflurane-anesthesia inhibited early-phase BBB disruption following cerebral ischemia when hypothermia was allowed, but not when the body temperature was maintained at ~37°C.

In addition to its inhibitory effect on ischemic BBB disruption, which is characterized by a marked extravasation of albumin and other macromolecular tracers, as reported in this study, isoflurane-anesthesia has also been previously reported to decrease the physiological extravasation of an amino acid [40]. Moreover, one study reported that isoflurane-anesthesia increases the extravasation of EBD when the tracer was injected at specific time points corresponding to a particular EEG signal [41]. In addition to isoflurane, non-gaseous anesthetics and analgesics also affect BBB permeability. Pentobarbital or ketamine, when administered alone, decreased BBB permeability to aminoisobutyric acid [42]. However, when combined with ethanol, pentobarbital (but not ketamine) injured cerebral endothelial cells, leading to an increase in the extravasation of intravascular horseradish peroxidase [43]. Like pentobarbital and ketamine, the opioid analgesic fentanyl also decreased BBB permeability to labeled aminoisobutyric acid [44]. However, in contrast with the *in vivo* effect, neither barbiturates nor fentanyl affected the permeability of aminoisobutyric acid, sucrose, or EBD-albumin through the brain microvascular endothelial cell monolayer in an *in vitro* culture system [45]. This result raised the intriguing possibility that, despite the lipophilic nature of these compounds, many of these anesthetics/analgesics did not affect BBB permeability by acting directly on the endothelial cell membrane.

BBB disruption can contribute to ischemic neuronal injury by introducing plasma proteases and inflammatory cells into the brain. Indeed, the degree of BBB disruption strongly correlates with the severity of neuronal injury [1], and some therapeutic agents that reduce ischemic BBB disruption also attenuate ischemic brain infarct volume [6, 7]. In contrast to this notion, we found that inhibiting BBB disruption at its peak during the early phase of cerebral ischemia (at 6 h post-ischemia), by a 1 h-long treatment of isoflurane-induced hypothermia, had no effect on the volume of cerebral infarction that was later determined at 24 h post-ischemia. In comparison, two previous studies reported a marked reduction of the cerebral infarction volume 24 h post-ischemia, following the injection of inhibitors of the neuronal death signaling cascades 6 h after the onset of ischemia [16, 17]. One of these studies used the same stroke model reported in the present study [17]. Altogether, these findings suggest that the inhibitors of the neuronal death signaling cascades may be more effective than inhibitors of BBB disruption as neuroprotective treatments for ischemic stroke. Similar to the inhibitors of the neuronal death signaling cascade, which are even more effective when administered immediately after a stroke [20], inhibiting the entire early-phase period of ischemic BBB disruption is likely to produce a better neuroprotective effect than inhibiting only the peak of BBB disruption. Therefore, our data do not exclude the possibility that the entire early phase of BBB disruption could contribute to ischemic brain infarction.
